# Circulating inflammatory cytokines influencing schizophrenia: a Mendelian randomization study

**DOI:** 10.3389/fpsyt.2024.1417213

**Published:** 2024-06-24

**Authors:** Yao-Ting Li, Xuezhen Zeng

**Affiliations:** ^1^ Department of Forensic Science, Guangdong Police College, Guangzhou, Guangdong, China; ^2^ Department of Pharmacy, The First Affiliated Hospital of Sun Yat-sen University, Guangzhou, Guangdong, China

**Keywords:** schizophrenia, inflammatory cytokines, Mendelian randomization, immunity, psychiatry disorder

## Abstract

**Introduction:**

Schizophrenia (SCZ) is a severe psychiatric disorder whose pathophysiology remains elusive. Recent investigations have underscored the significance of systemic inflammation, particularly the impact of circulating inflammatory proteins, in SCZ.

**Methods:**

This study explores the potential causal association between certain inflammatory proteins and SCZ. Bidirectional Mendelian randomization (MR) analyses were conducted utilizing data from expansive genome-wide association studies (GWAS). Data regarding circulating inflammatory proteins were sourced from the GWAS Catalog database, encompassing 91 inflammatory cytokines. SCZ-related data were derived from the Finngen database, incorporating 47,696 cases and 359,290 controls. Analytical methods such as inverse variance weighted, MR-Egger, weighted median, simple mode, and weighted mode were employed to evaluate the association between inflammatory cytokines and SCZ. Sensitivity analyses were also performed to affirm the robustness of the results.

**Results:**

Following FDR adjustment, significant associations were observed between levels of inflammatory cytokines, including Fibroblast Growth Factor 5 (OR = 1.140, 95%CI = 1.045, 1.243, *p* = 0.003, FDR=0.015), C-C Motif Chemokine 4 (OR = 0.888, 95%CI = 0.816, 0.967, *p* = 0.006, FDR = 0.015), C-X-C Motif Chemokine 1 (OR = 0.833, 95%CI = 0.721, 0.962, *p* = 0.013, FDR = 0.064), and C-X-C Motif Chemokine 5 (OR = 0.870, 95%CI = 0.778, 0.973, *p* = 0.015, FDR = 0.074), and the risk of SCZ.

**Conclusion:**

Our results from MR analysis suggest a potential causal link between circulating inflammatory cytokines and SCZ, thereby enriching our understanding of the interactions between inflammation and SCZ. Furthermore, these insights provide a valuable foundation for devising therapeutic strategies targeting inflammation.

## 1 Introduction

Schizophrenia, a pervasive and debilitating psychiatric disorder, affects approximately 1% of the global population and significantly impacts public health as a leading cause of disability and premature mortality (1). This disorder is characterized by a varied spectrum of cognitive, behavioral, and emotional disturbances, devoid of a singular defining symptom. It involves positive symptoms, such as hallucinations and delusions, as well as negative symptoms, including reduced emotional expression, diminished motivation, and social withdrawal.

From an epidemiological standpoint, overwhelming evidence indicates that schizophrenia’s heritability is substantial, with estimates reaching as high as 81% based on extensive twin and family studies (2). The field of genetic epidemiology has experienced remarkable evolution, initially engaging in linkage studies and candidate gene assessments, progressing to genome-wide association studies (GWAS), and currently adopting next-generation sequencing (NGS) methodologies (3). GWAS, in particular, have broadened participant pools and enhanced statistical capabilities to identify common genetic variants linked to schizophrenia. These investigations have identified thousands of unique genetic sites that, together, contribute to a segment of the disorder’s heritability, as represented by single nucleotide polymorphisms (SNPs).

Inflammatory cytokines are central to the pathophysiology of schizophrenia, contributing significantly to neuroinflammatory processes. These cytokines modulate neuronal receptor activity, affecting neuronal communication and the dynamics of neurotransmitter release and re-uptake. Research has revealed variations in particular inflammatory cytokines in those with schizophrenia, including changes in levels of interleukins (IL) and tumor necrosis factor (TNF) (4). Such variations may be closely related to the genesis of schizophrenia and imply a potential link between irregular cytokine levels and the development of the disease.

IL-6 is one of the most extensively studied cytokines in schizophrenia research to date. Levels of IL-6 in both peripheral blood and cerebrospinal fluid are elevated across all patient groups (including those with first-episode psychosis, clinical high risk or ultra-high risk for psychosis) compared to healthy controls (5–7). Evidence suggests that the variability of IL-6 levels is significantly reduced compared to healthy controls, indicating that elevated IL-6 levels may be a key factor in the immune phenotype of schizophrenia (8). Concentrations of IL-6 in the blood are positively correlated with both negative and positive symptoms, as well as overall psychopathological manifestations and cognitive impairments (9, 10).

Furthermore, numerous studies have found elevated levels of IL-1β in drug-naïve first-episode psychosis patients (11), first-episode psychosis patients in both adults and children (most of whom were on atypical antipsychotic medications (12–14)), and in stable, acutely relapsed, or recovering chronic patients (15, 16). The levels of interleukin factors appear to be associated with the course of the disease and the severity of symptoms. Patients with elevated levels of IL-6, IL-8, and IL-4 tend to have a longer duration of illness and longer hospital stays (17, 18). Moreover, higher levels of IL-6, IL-1β, IL-33, and IL-17 are associated with more severe positive symptoms (19–22). Moreover, as potential biomarkers, inflammatory cytokines provide insight into the clinical state and treatment response of patients. Recent studies indicate that cytokine fluctuations are associated with symptom severity, disease progression, and responsiveness to antipsychotic treatment in schizophrenia. Therefore, cytokines represent promising indicators for early schizophrenia detection, disease monitoring, and treatment outcome assessment (23).

In this study, we perform a comprehensive two-sample Mendelian randomization (MR) analysis to elucidate the causal relationship between 91 inflammation-related biomarkers and the incidence of schizophrenia. Our study rests on three crucial assumptions: 1) the genetic variation selected as the instrumental variable (IV) is genuinely correlated with the exposure of interest; 2) the genetic variation must not be related to any confounding factors; and 3) the genetic variation affects the outcome only through exposure. The objective of this analysis is to uncover the inflammatory underpinnings of schizophrenia and identify potential targets for intervention that may slow down the progression and improve patient outcomes.

## 2 Methods

### 2.1 Study design

**Figure 1:**
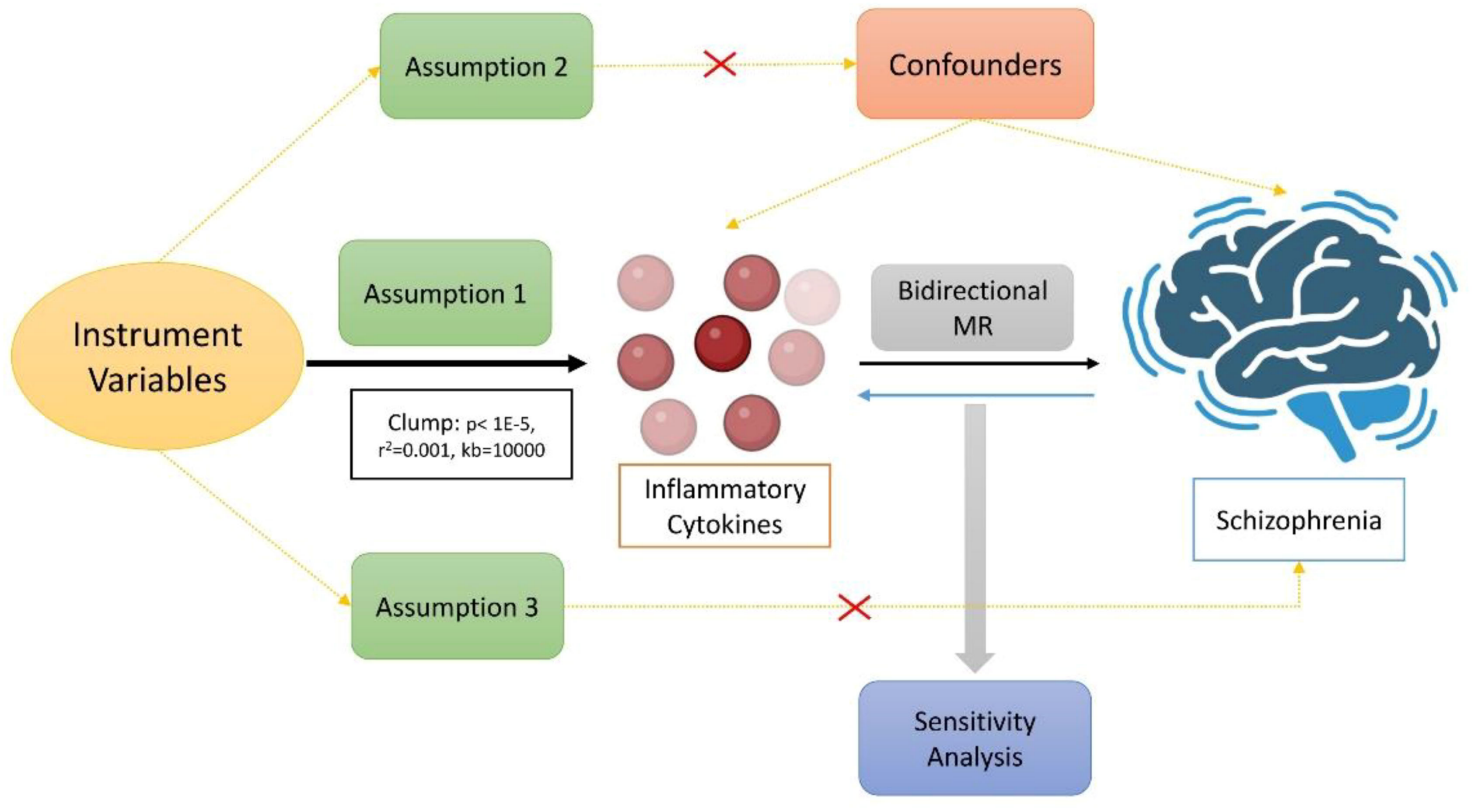
The main design of the causal relationship between inflammatory cytokines and schizophrenia through directional MR analyses.

Through bidirectional two-sample MR analysis, we examined the causal link between 91 inflammatory cytokines and SCZ. Our approach was based on three core hypotheses: first, instrument variables (IVs) were robustly linked to exposure factors; second, IVs remained unaffected by confounders, whether known or unknown; and third, IVs influenced the outcome factors solely via exposure factors (24). **Figure 1** depicts the comprehensive study design. The data leveraged in our research originated from the OPEN GWAS public databases and the Finngen database and it underwent anonymization before publication to ensure the absence of personal or identifiable information.

### 2.2 Data source

The GWAS Catalog, containing entries from GCST90274758 to GCST90274848, provides a comprehensive repository of GWAS statistics for each circulating inflammatory protein (25). The catalog includes extensive data derived from 14,824 European individuals, covering 91 inflammatory cytokines. In selecting schizophrenia-related data from the Finnish database (https://www.finngen.fi/en), we considered factors such as sample size, publication year, the number of SNPs, and ethnicity. The chosen datasets comprise 406,986 participants of European descent, including 47,696 cases and 359,290 controls (**Supplementary Table 1**).

### 2.3 Selection of instrumental variables

The cutoff value of 1×10–5 was applied in independent and significant SNPs for each immune trait. Linkage disequilibrium (LD) analysis was conducted using a rigorous threshold (r^2^ < 0.001) across a 10,000 kb range. To ensure the effect alignment, SNPs were harmonized across exposure and outcome datasets based on allele type. The F-statistic was calculated as F = R^2^ × (N-2)/(1-R^2^), with R2 denoting the extent to which the IVs explain the exposure factors and N denoting the sample size of the exposed GWAS data (26). SNPs with an F-statistic less than 10, suggestive of potential weak instrumental variable bias, were removed to mitigate impact on analyses. Subsequently, the resultant SNPs that corresponded with hypotheses were extracted from databases such as IEU OpenGWAS or FinnGen, and associations were ascertained. The final set of SNPs served as the definitive IVs for exposure.

### 2.4 MR analyses

MR analyses was performed using a variety of approaches: inverse-variance weighted (IVW), weighted mode, simple mode, weighted median, and MR Egger. The IVW method, utilized as the primary MR technique, integrates the Wald ratios of individual SNPs using meta-analysis. It operates under the assumption that IVs influence the outcomes solely through the exposure, which should yield unbiased causal estimates if horizontal pleiotropy is absent (27). Thus, we utilized the IVW method to provide reliable estimation in our study. To enhance our analytical framework and pinpoint potential biases stemming from ineffective IVs or horizontal pleiotropy, we employed the weighted median and MR-Egger methods. Nevertheless, results from these methods might be less precise due to the susceptibility to the influence of outlier genetic variants, particularly those from the MR-Egger method (28). While the weighted median approach presents lesser bias, it suffers from reduced precision. Despite their lower efficiency, the MR-Egger, simple mode, weighted mode, and weighted median methods were applied in supplementary analyses. This study complies with the Strengthening the Reporting of Observational Studies in Epidemiology using Mendelian Randomization (STROBE-MR) guidelines, as demonstrated by the checklist included in the **Supplementary Material** (**Supplementary Table 7**).

### 2.5 Sensitivity analysis

A sensitivity analysis was conducted to evaluate potential heterogeneity and pleiotropy. Firstly, IVW and MR-Egger regression were used, and Cochran Q statistics were generated to quantify heterogeneity. Furthermore, the intercept from MR-Egger regression was utilized to investigate horizontal pleiotropy (29). Leave-one-out analysis was carried out to determine the influence of excluding individual SNPs on the aggregate results (30). Scatter plots verifying the horizontal pleiotropy of inflammatory cytokines causally associated with SCZ. Additionally, funnel plots ascertained the integrity of the correlations and confirmed the absence of heterogeneity. All statistical procedures were executed with R software, version 4.3.1. MR analysis was performed employing the “TwoSampleMR” package in R.

## 3 Result

### 3.1 Genetic prediction of circulating inflammatory cytokines for schizophrenia risk

**Figure 2:**
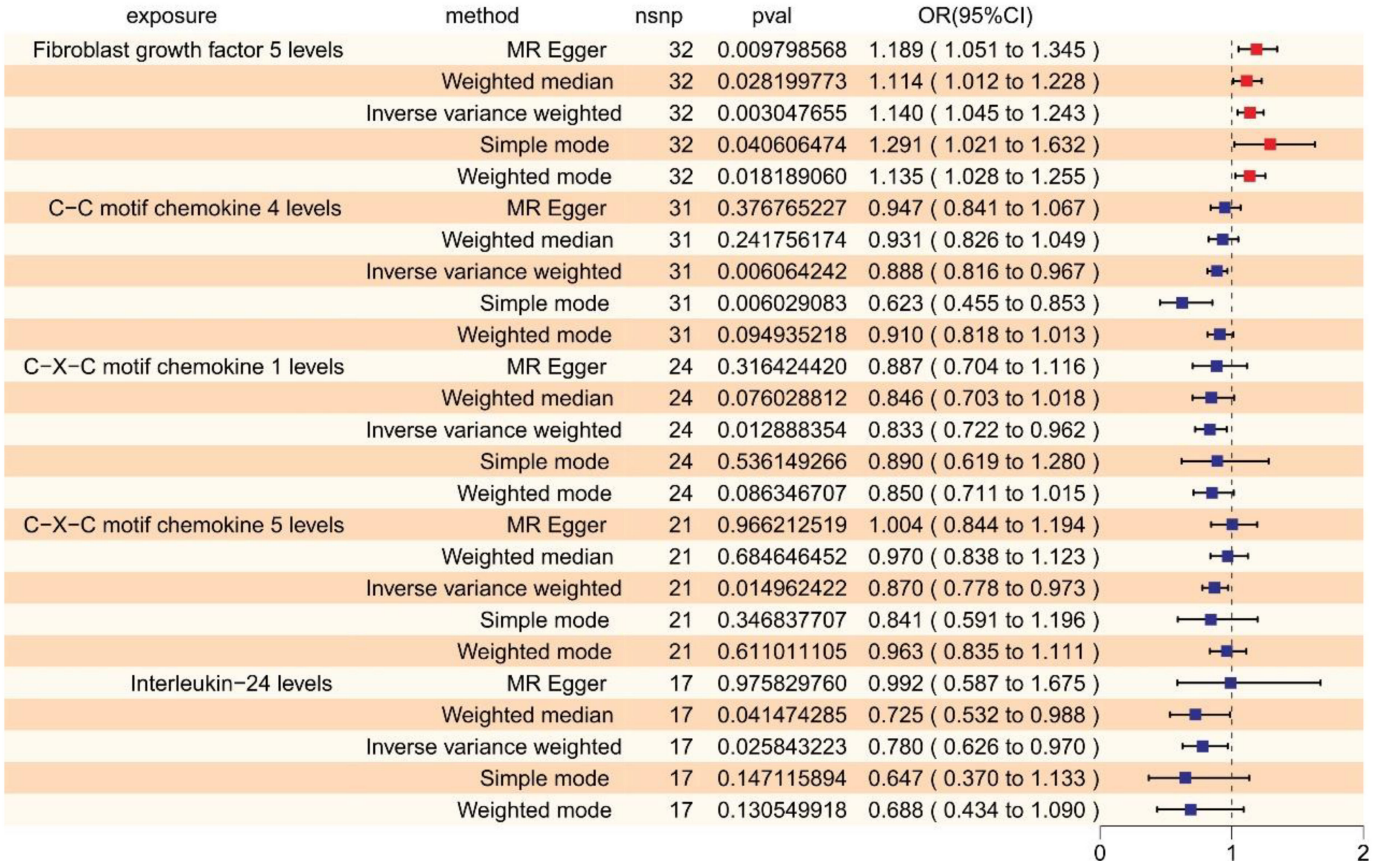
The forest plot shows the causal associations between inflammatory cytokines and SCZ by forward MR, we mainly used the IVW method. OR < 1 indicates a negative association between exposure and outcome, a noteworthy point that is represented in blue within the graphical data. While OR > 1 indicates a positive association between exposure and outcome, a noteworthy point that is represented in red within the graphical data.

Applying a relatively relaxed threshold (p < 1×10–5), we identified independent SNPs for all 91 inflammatory cytokines. Within this scope, 13 to 47 SNPs reached genome-wide significance as IVs for inflammatory cytokines, with F-statistics ranging markedly from 19.51 to 3468.99 (**Supplementary Table 6**). We found that Fibroblast Growth Factor 5 (FGF5) had an odds ratio (OR) of 1.140 (95% CI: 1.045–1.243, *p* = 0.003, False Discovery Rate (FDR) = 0.015) according to the IVW method, an OR of 1.189 (95% CI: 1.051–1.345, *p =* 0.0098) by MR-Egger, an OR of 1.114 (95% CI: 1.012–1.228, *p =* 0.028) by weighted median, an OR of 1.291 (95% CI: 1.021–1.632, *p =* 0.041) by simple mode, and an OR of 1.135 (95% CI: 1.028–1.255, *p =* 0.018) by weighted mode. For C-C Motif Chemokine 4 (CCL4), the IVW method yielded an OR of 0.888 (95% CI: 0.816–0.967, *p =* 0.006, FDR = 0.015), while the MR-Egger, weighted median, simple mode, and weighted mode indicated an OR of 0.947 (95% CI: 0.841–1.067, *p =* 0.377), 0.931 (95% CI: 0.826–1.049, *p =* 0.242), 0.623 (95% CI: 0.455–0.853, *p =* 0.006), and 0.910 (95% CI: 0.818–1.013, *p =* 0.095) respectively. Regarding C-X-C Motif Chemokine 1 (CXCL1), the IVW method estimated an OR of 0.833 (95% CI: 0.721–0.962, *p =* 0.013, FDR = 0.064). Results from additional methods were as follows: MR-Egger with an OR of 0.887 (95% CI: 0.704–1.116, *p =* 0.316), weighted median at an OR of 0.846 (95% CI: 0.703–1.018, *p =* 0.076), simple mode with an OR of 0.890 (95% CI: 0.619–1.280, *p =* 0.536), and weighted mode at an OR of 0.850 (95% CI: 0.711–1.015, *p =* 0.086). In the case of C-X-C Motif Chemokine 5 (CXCL5), the IVW method calculated an OR of 0.870 (95% CI: 0.778–0.973, *p =* 0.015, FDR = 0.074). Alternative approach results were: MR-Egger at an OR of 1.004 (95% CI: 0.844–1.194, *p =* 0.966), weighted median with an OR of 0.970 (95% CI: 0.838–1.123, *p =* 0.685), simple mode reporting an OR of 0.841 (95% CI: 0.591–1.120, *p =* 0.347), and weighted mode showing an OR of 0.963 (95% CI: 0.835–1.111, *p =* 0.611). For Interleukin-24 (IL-24), an OR of 0.780 (95% CI: 0.626–0.970, *p =* 0.026, FDR = 0.103) was determined by the IVW method. Despite the P value for IL-24 being less than 0.05, its FDR corrected value exceeds the threshold of 0.1. The findings from other methods included: MR-Egger with an OR of 0.992 (95% CI: 0.587–1.675, *p =* 0.976), weighted median at an OR of 0.725 (95% CI: 0.532–0.988, *p =* 0.041), simple mode yielding an OR of 0.647 (95% CI: 0.370–1.133, *p =* 0.147), and weighted mode indicating an OR of 0.688 (95% CI: 0.434–1.090, *p =* 0.131). These results are visualized in **Figure 2** and detailed in **Supplementary Table 2**.

### 3.2 Genetic prediction of schizophrenia for risk of circulating inflammatory cytokines

**Figure 3:**
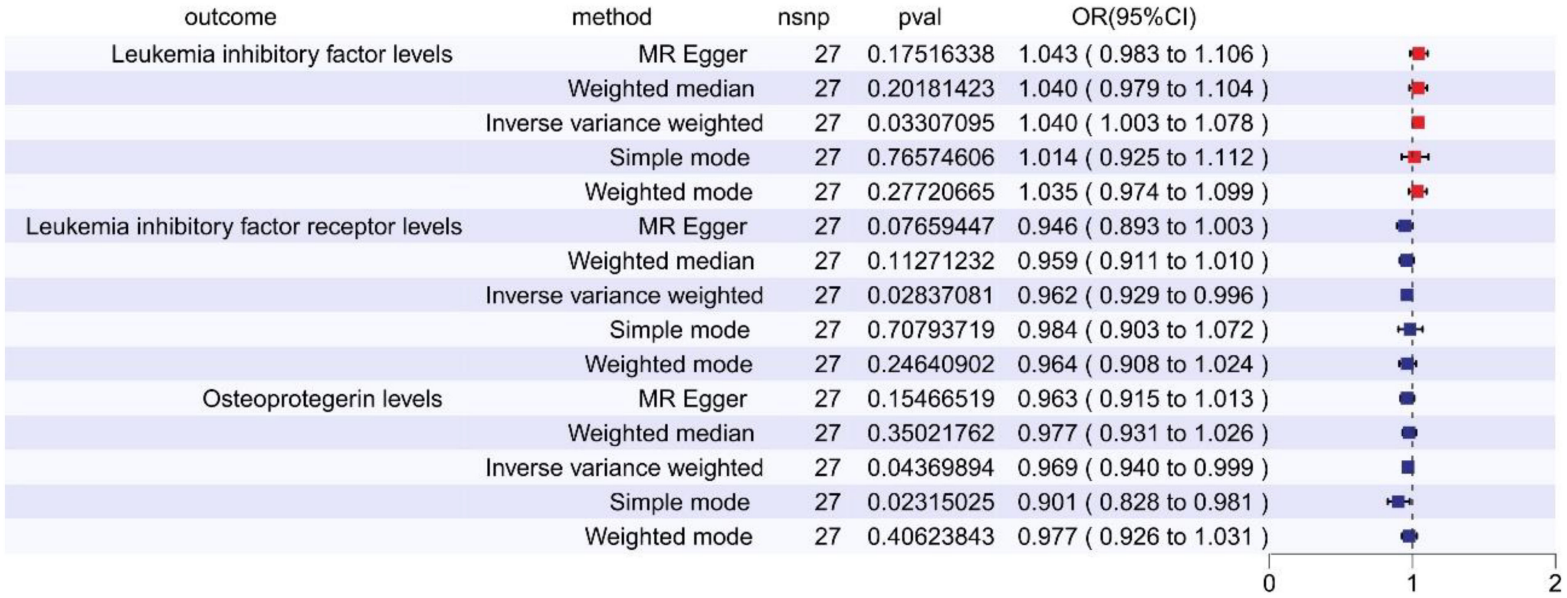
The forest plot shows the causal associations between inflammatory cytokines and SCZ by reverse MR, we mainly used the IVW method. OR < 1 indicates a negative association between exposure and outcome, a noteworthy point that is represented in blue within the graphical data. While OR > 1 indicates a positive association between exposure and outcome, a noteworthy point that is represented in red within the graphical data.

In the reverse MR analysis examining the relationship between SCZ and 91 inflammatory cytokines, we observed the following outcomes: Elevated levels of Leukemia Inhibitory Factor (LIF) were linked to an increased risk of schizophrenia (OR = 1.040, 95% CI: 1.003–1.078, *p =* 0.033, FDR = 0.165, via IVW). Conversely, reduced levels of Leukemia inhibitory factor receptor (LIF-R) were associated with an augmented risk of schizophrenia (OR = 0.962, 95% CI: 0.929–0.996, *p =* 0.028, FDR = 0.142, via IVW). Likewise, lower levels of Osteoprotegerin (OPG) correlated with a heightened risk of schizophrenia (OR = 0.969, 95% CI: 0.940–0.999, *p =* 0.044, FDR = 0.109, via IVW) (refer to **Figure 3**, **Supplementary Table 3**). These relationships exhibited neither significant heterogeneity nor evidence of horizontal pleiotropy. However, after FDR adjustment, none of these cytokines remained statistically significant.

### 3.3 Sensitivity analysis for the causal relationship between inflammatory cytokines and Schizophrenia

To confirm the causal link between inflammatory cytokines and schizophrenia, we conducted sensitivity analyses and detailed the results in the **Supplementary Material** (**Supplementary Tables 4**, **5**). Q values from MR-Egger and IVW tests indicated no substantial heterogeneity in any significant correlations (all p > 0.05). Additionally, we utilized the MR-Egger intercept and the MR-PRESSO Global test to investigate pleiotropy. Any horizontal pleiotropic outliers identified by MR-PRESSO were removed before re-analysis. Consequently, no evidence of horizontal pleiotropy was found in the association between any inflammatory cytokines and SCZ (all p > 0.05). Subsequent leave-one-out analyses revealed that no individual SNP predominantly influenced the relationship between inflammatory cytokines and SCZ. We illustrated these findings with scatter plot, funnel pot and leave-one analysis (**Supplementary Figures S1**–**S3**). Likewise, in reverse analyses, no specific SNP substantially altered the results, as depicted in **Supplementary Figures S4**, **S5**.

## 4 Discussion

In this study, we utilized forward and reverse MR analyses which enable an extensive examination of the potential bidirectional causal relationship between inflammatory cytokines and SCZ. The forward MR analysis identified that enhanced levels of particular inflammatory cytokines correlated with a higher risk of SCZ. Conversely, the reverse MR analysis indicated that an elevated risk of SCZ contributed to alterations in the concentrations of certain inflammatory cytokines. These results imply a potential two-way causal relationship, adding complexity to the interplay between inflammatory cytokines and SCZ.

Numerous prior studies have reported that expression levels of inflammatory cytokines play pivotal roles in the pathogenesis and prognosis of SCZ (31). Typically, malignant alterations in SCZ showed abrupt escalations or reductions in the expression levels of these cytokines. Thus, in this study, we intent to profile cytokine and monitor of their expression dynamics to understand the causal relationship between their levels and risk of SCZ. Through screening cytokine data, we initially discovered that abnormal expression of FGF5 might elevate the risk of SCZ. Large-scale genetic association studies have linked SNPs near the FGF gene locus with the emergence of SCZ (32, 33). Additionally, SNPs in the Fibroblast Growth Factor Receptor 1 (FGFR) gene region have demonstrated a statistical correlation with SCZ (34), hinting at possible functional disruptions in the FGF signaling pathway during the development of this disorder. Moreover, postmortem examinations of brain tissues from SCZ patients have revealed variations in FGFR mRNA expression within the hippocampus and additional cerebral regions (35), suggesting FGF signaling dysregulation in individuals diagnosed with SCZ.

Furthermore, we also found that increased cytokine levels may exert a protective effect against SCZ. Our results showed that the expression of CXCL1 have changed in SCZ. Previous study has demonstrated that the upregulation of CXCL1 in certain brain regions, particularly where demyelination occurs, enhanced the growth of oligodendrocyte precursor cells. Then, these oligodendrocyte precursor cells developed into oligodendrocytes that contribute to remyelination and potentially inhibit the progression of the disease (36). Moreover, CXCL1 has the capacity to attract neutrophils to the brain, which partially inhibit autoreactive T cell activities, thus slowing SCZ exacerbation (37). In addition to CXCL1, we also identified substantial associations between the risk of SCZ and fluctuations in the levels of various inflammatory proteins, including CCL4, CXCL5, and IL-24. These findings suggest that these proteins might exert protective effects in the context of SCZ.

In the reverse MR analysis, we observed that an increased risk of SCZ contributed to altered levels of specific inflammatory proteins, including LIF and its receptor (LIF-R), and OPG. The LIF gene resides at chromosomal region 22q12.1-q12.2, and has been identified as a susceptibility locus for SCZ. Substantial evidence links the LIF gene and its SNPs with an increased risk of SCZ. And particular variants of the LIF gene are significantly associated with the diagnosis of schizophrenia, especially in patients with hebephrenic SCZ. LIF also plays a role in the normal inflammatory responses of the central and peripheral nervous systems (38), influencing the activity of astrocytes, oligodendrocytes, microglia, and immune cells. Accumulating studies have identified alterations in the expression levels of LIF and its related cytokines in patients with SCZ, which indicates an augmented neuroinflammatory state that indirectly impacts the disease’s pathological progression (39). Moreover, antipsychotic drugs like chlorpromazine can stimulate the LIF-R, affecting serum LIF receptor levels (9). Unfortunately, following the adjustment for FDR, the statistical significance of these cytokines was not maintained. Additional MR investigations are warranted to validate our results as more extensive GWAS datasets become available.

## 5 Limitations

This study presents potential limitations, with the primary constraint being the reliance on GWAS summary datasets for inflammatory protein traits and SCZ, despite these being the most comprehensive datasets available. Variations in sample size, quality control methodologies, and the ethnic diversity of the study populations could introduce potential biases. The datasets utilized in this research are exclusively derived from European cohorts, and the employment of summary-level data precludes the possibility of individual-level analysis. To provide a more comprehensive perspective, future Mendelian Randomization studies utilizing individual-level data are necessary. This limitation hampers our ability to conduct stratified analyses by demographic variables such as race, gender, and age. Furthermore, despite the application of stringent criteria for linkage disequilibrium pruning (r2 < 0.001, distance > 10,000 kb), the selection of SNPs under a less stringent significance threshold (p < 1×10^-5^) was necessitated due to the limited number of SNPs achieving genome-wide significance. However, the F-statistics for each SNP were greater than 10, indicating the absence of weak instrument bias. We employed a Benjamini-Hochberg FDR threshold of 0.1 to identify results of significance. This FDR approach, while less conservative than the Bonferroni method, offers a more practical balance between the risk of false positives and the pursuit of novel findings. Future larger-scale GWAS are required to identify more genetic loci associated with inflammatory proteins.

## 6 Conclusion

In summary, our study has revealed associations between four inflammatory cytokines and the risk of SCZ. Specifically, we highlight FGF5 as a potential pathogenic risk-enhancing inflammatory factor, while CCL4, CXCL1, and CXCL5 are posited as protective candidate inflammatory cytokines for SCZ. Our findings offer new insights into the inflammatory cytokines to reveal the pathogenesis and therapeutic strategies for SCZ. Focusing on FGF5 as a therapeutic target may herald a significant advancement in the treatment of SCZ, paving the way for more effective and tailored therapeutic approaches. To fully understand the potential roles of these inflammatory cytokines and the corresponding drug targets in the development and progression of SCZ, further studies with larger sample sizes and more comprehensive genetic coverage are warranted, taking into account other potential confounding factors. Future investigations should concentrate on corroborating these discoveries and examining the interactions among additional cytokines and SCZ.

## Data availability statement

The original contributions presented in the study are included in the article/**Supplementary Material**, further inquiries can be directed to the corresponding author.

## Ethics statement

Ethical approval was not required for the study involving humans in accordance with the local legislation and institutional requirements. Written informed consent to participate in this study was not required from the participants or the participants’ legal guardians/next of kin in accordance with the national legislation and the institutional requirements.

## Author contributions

YL: Writing – original draft, Writing – review & editing. XZ: Writing – original draft, Writing – review & editing.
