# Supplementary material for: Circulating inflammatory cytokines influencing schizophrenia: a Mendelian randomization study
Source: Front Psychiatry. 2024 Jun 24;15:1417213. doi: 10.3389/fpsyt.2024.1417213 (PMC11228335; doi:10.3389/fpsyt.2024.1417213)

Supplementary Figures

Figure S1: The Scatter plot of the forward MR between inflammatory cytokines and SCZ.

Figure S2: The funnel plot of the forward MR analysis.

Figure S3: Leave-one-out plot of sensitivity analysis for the forward MR analysis.

Figure S4: The Scatter plot and funnel plot of the reverse MR analysis.

Figure S5: Leave-one-out plot of sensitivity analysis for the reverse MR analysis.

Figure S1: Scatter plot visualized the forward MR of the effects. The x-axis is the size of the SNP effect of inflammatory cytokines, and the y-axis is the size of the SNP effect of the SCZ. Different MR methodologies are distinguished by unique color coding (A: CCL4, B: CXCL1, C: CXCL5, D: FGF5, E: IL-24).


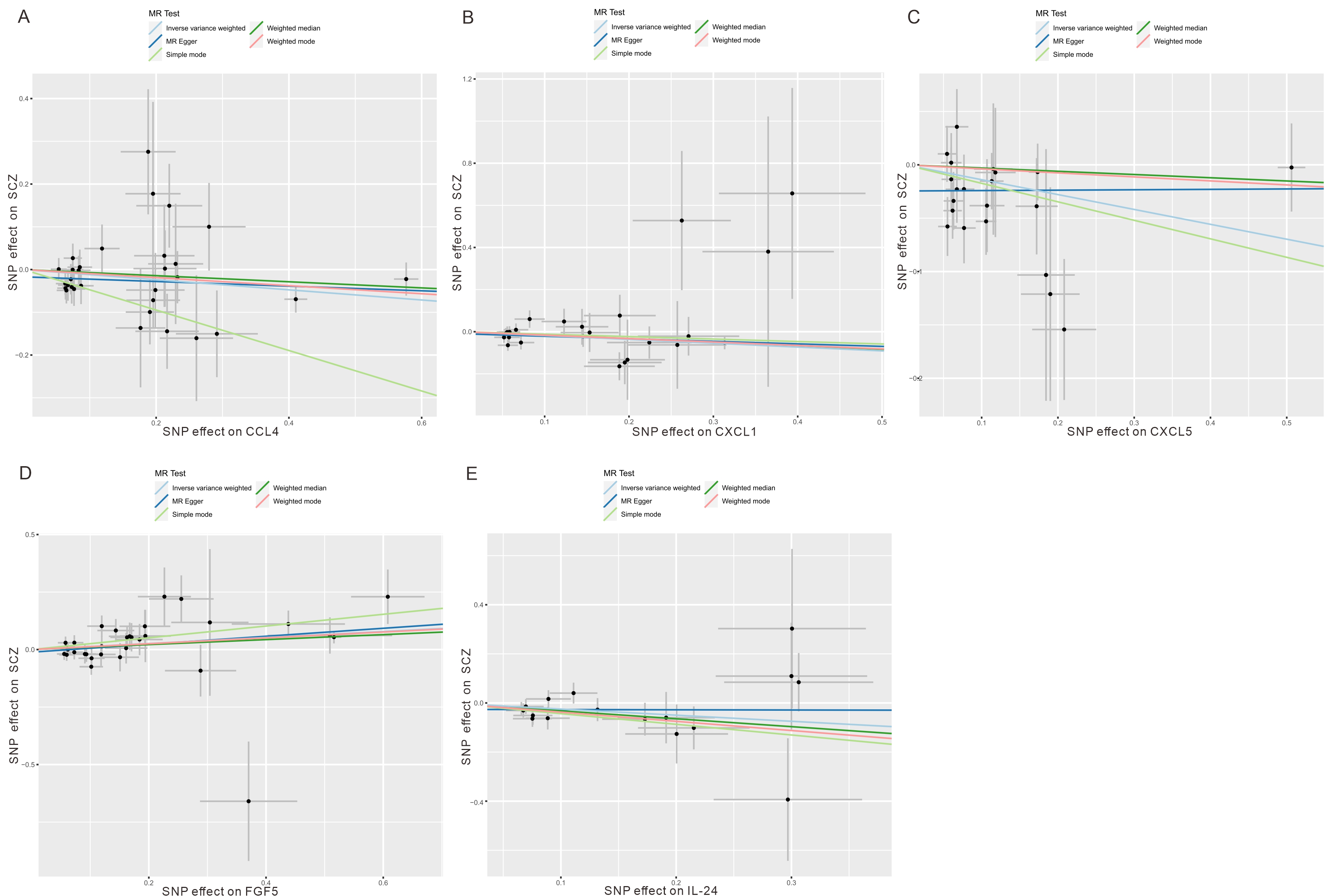


Figure S2: The funnel plot is utilized to evaluate the bias and heterogeneity for the positive results with inflammatory cytokines as exposures and SCZ as outcomes (A: CCL4, B: CXCL1, C: CXCL5, D: FGF5, E: IL-24).


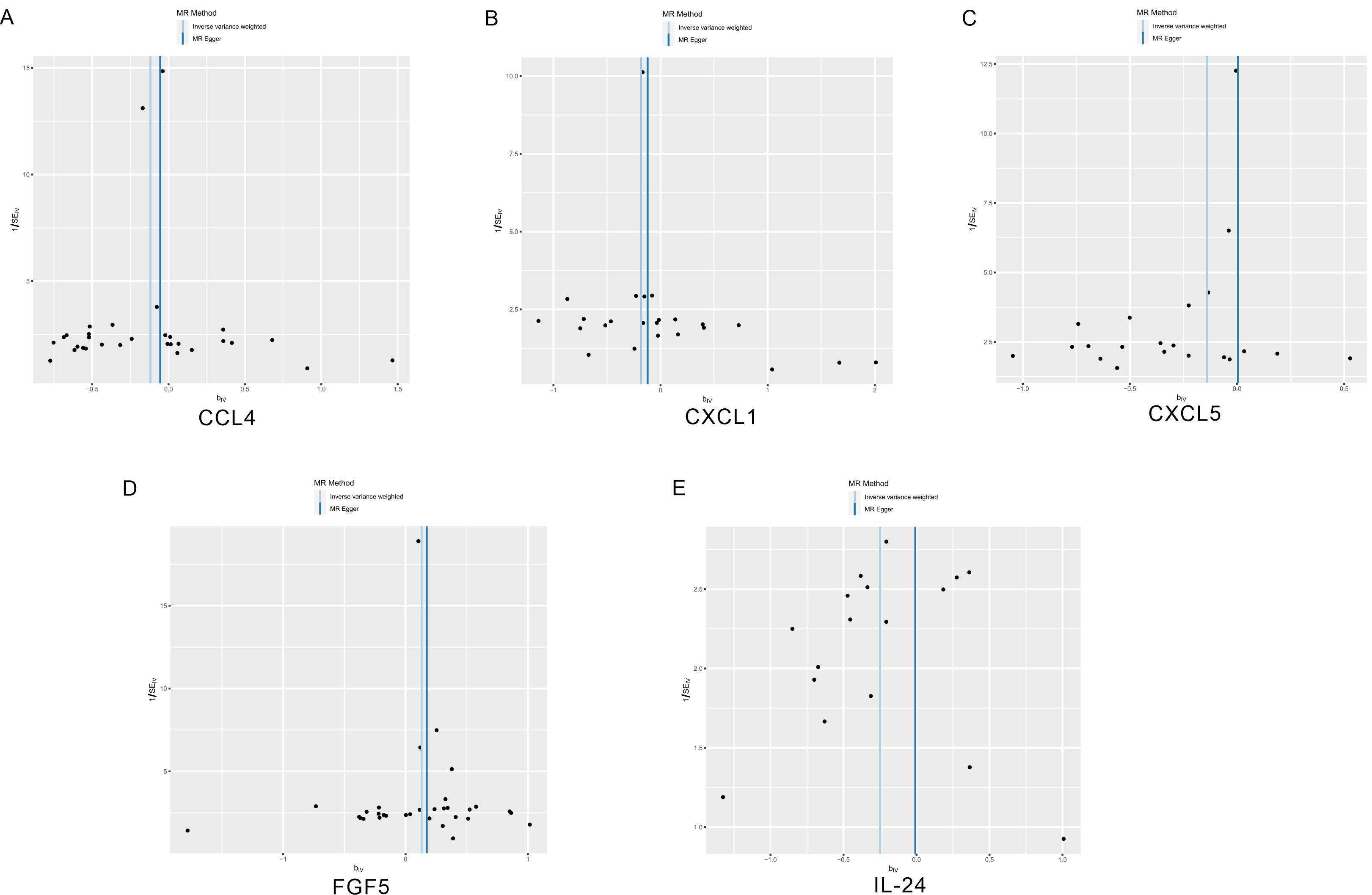


Figure S3: Leave-one-out stability tests of the univariable forward mendelian randomization analyses for inflammatory cytokines on SCZ. Calculate the MR results of the remaining IVs after removing the IVs one by one (A: CCL4, B: CXCL1, C: CXCL5, D: FGF5, E: IL-24).


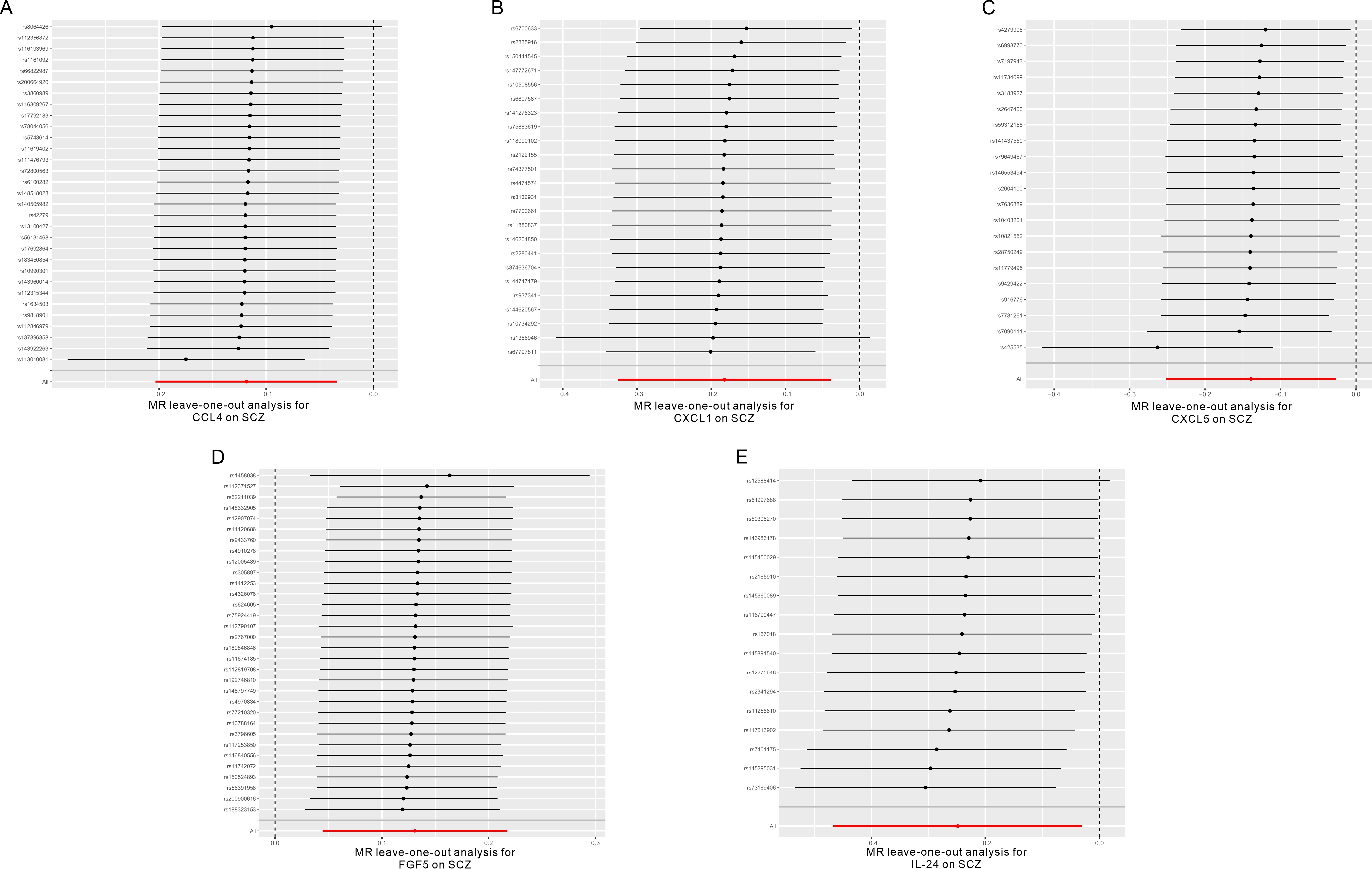


Figure S4: Scatter plot visualized the reverse MR of the effects. The x-axis is the size of the SNP effect of the SCZ, and the y-axis is the size of the SNP effect of inflammatory cytokines. Different MR methodologies are distinguished by unique color coding (A: LIF, B: LIF-R, C: OPG). The funnel plot is utilized to evaluate the bias and heterogeneity for the positive results with SCZ as exposures and inflammatory cytokines as outcomes (D: LIF, E: LIF-R, F: OPG).


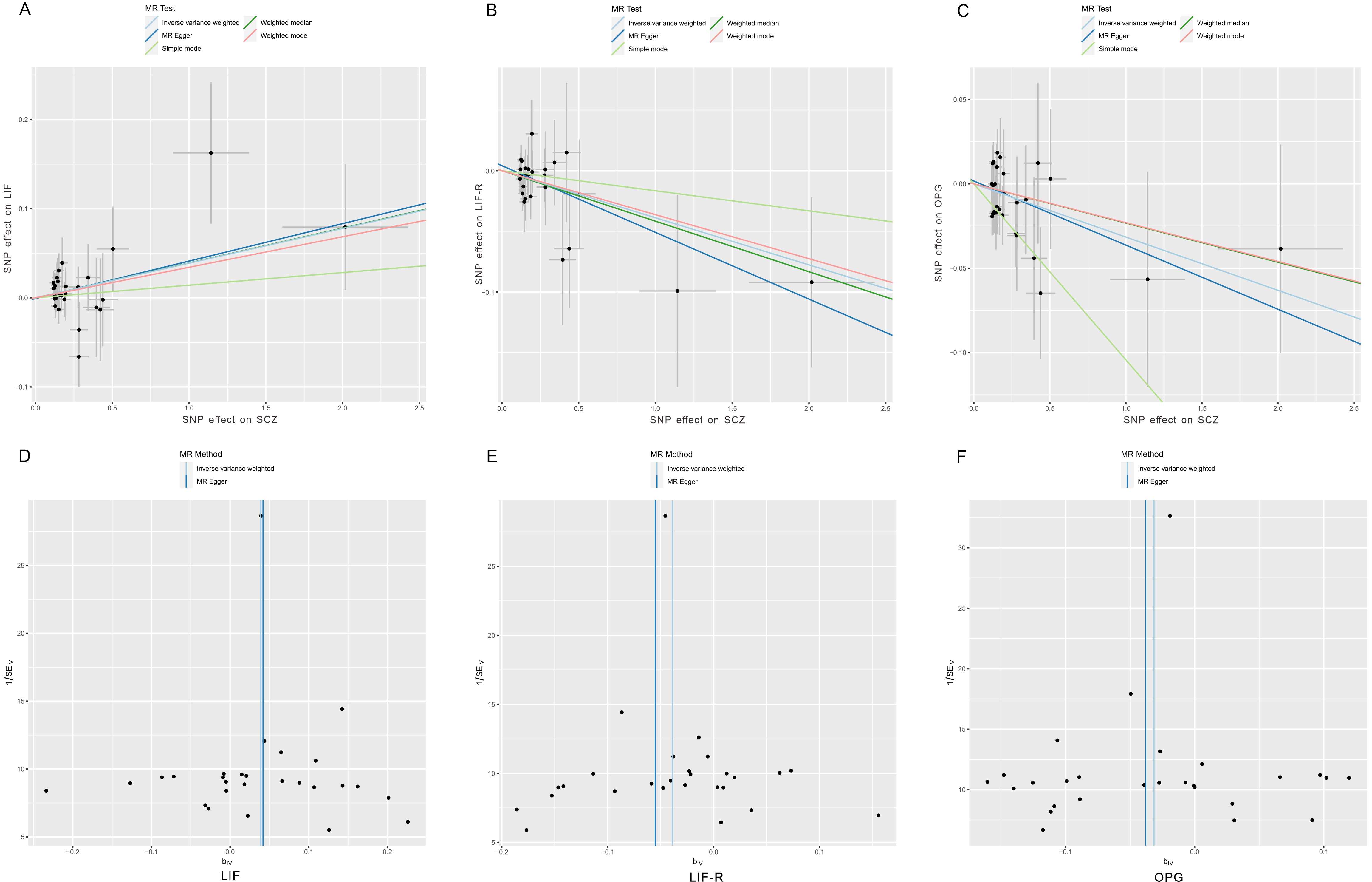


Figure S5: Leave-one-out stability tests of the univariable reverse mendelian randomization analyses for SCZ on inflammatory cytokines. Calculate the MR results of the remaining IVs after removing the IVs one by one (A: LIF, B: LIF-R, C: OPG).


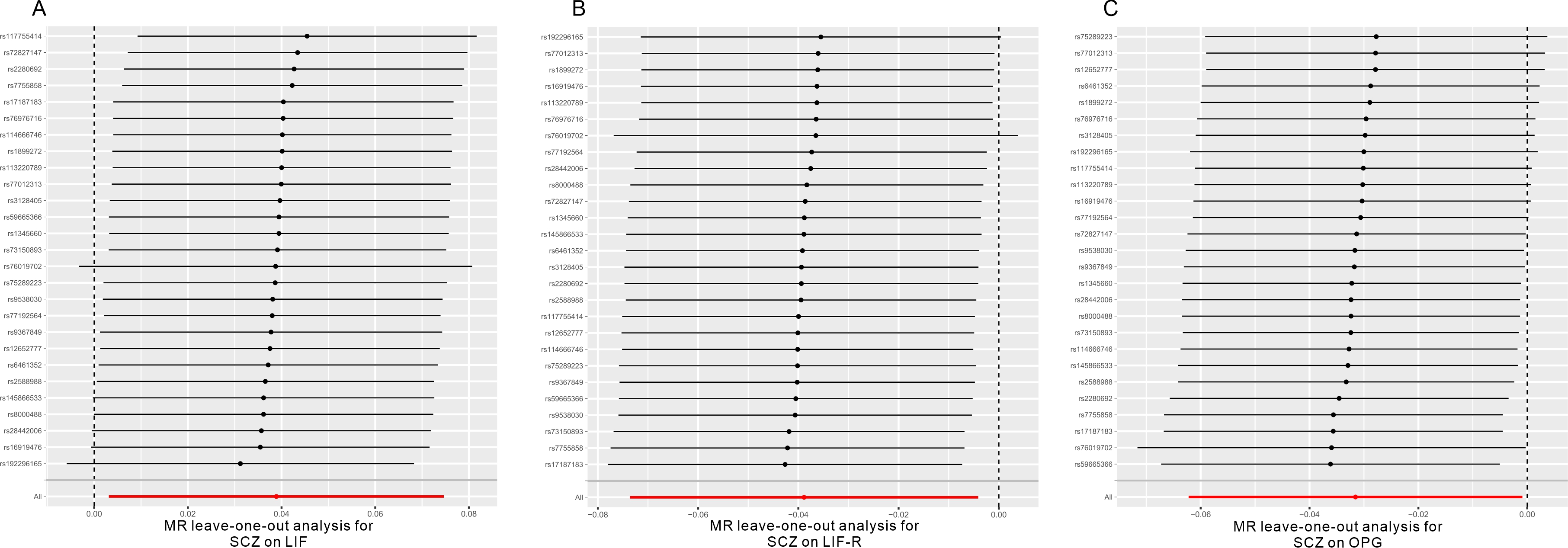

Supplement: Supplementary file 1 [file Table_1.docx]
